# The effect of increasing temperature on crop photosynthesis: from enzymes to ecosystems

**DOI:** 10.1093/jxb/erab090

**Published:** 2021-02-23

**Authors:** Caitlin E Moore, Katherine Meacham-Hensold, Pauline Lemonnier, Rebecca A Slattery, Claire Benjamin, Carl J Bernacchi, Tracy Lawson, Amanda P Cavanagh

**Affiliations:** 1 School of Agriculture and Environment, The University of Western Australia, Crawley, Australia; 2 Institute for Sustainability, Energy & Environment, University of Illinois at Urbana-Champaign, Urbana, USA; 3 Center for Advanced Bioenergy and Bioproducts Innovation, University of Illinois at Urbana-Champaign, Urbana, USA; 4 Carl R. Woese Institute for Genomic Biology, University of Illinois at Urbana-Champaign, Urbana, USA; 5 School of Life Sciences, University of Essex, Colchester, UK; 6 Global Change and Photosynthesis Research Unit, United States Department of Agriculture–Agricultural Research Service, Urbana, USA; 7 Department of Crop Sciences, University of Illinois at Urbana-Champaign, Urbana, USA; 10 The James Hutton Institute, UK

**Keywords:** Cropping system, gross primary productivity, heat stress, resilience, Rubisco, stomata, vapour pressure deficit

## Abstract

As global land surface temperature continues to rise and heatwave events increase in frequency, duration, and/or intensity, our key food and fuel cropping systems will likely face increased heat-related stress. A large volume of literature exists on exploring measured and modelled impacts of rising temperature on crop photosynthesis, from enzymatic responses within the leaf up to larger ecosystem-scale responses that reflect seasonal and interannual crop responses to heat. This review discusses (i) how crop photosynthesis changes with temperature at the enzymatic scale within the leaf; (ii) how stomata and plant transport systems are affected by temperature; (iii) what features make a plant susceptible or tolerant to elevated temperature and heat stress; and (iv) how these temperature and heat effects compound at the ecosystem scale to affect crop yields. Throughout the review, we identify current advancements and future research trajectories that are needed to make our cropping systems more resilient to rising temperature and heat stress, which are both projected to occur due to current global fossil fuel emissions.

## Teaser

This review explores current understanding in crop photosynthesis and temperature across scales, linking enzyme processes within the leaf to stomata, plant transport systems, individual plants, and ecosystem-scale responses.

## Introduction

Global land surface temperatures are increasing due to rising atmospheric CO_2_ from anthropogenic emissions that are causing climate change, and with this comes the challenge of meeting food and fuel supply demands under more stressful crop growing conditions. Despite a drop in emissions associated with the coronavirus pandemic of 2020 (COVID-19; Le Quéré *et al.*, 2020), global emissions are currently tracking the worst-case ‘business as usual’ emissions scenario (RCP8.5) that will very likely equate to unprecedented warming from pre-industrial (1850–1990) levels of 3–5 °C by 2100 (IPCC, 2014). A recent IPCC report indicated, with medium confidence, that crop yields will experience ‘severe and widespread impacts’ if global warming exceeds 1.5 °C above pre-industrial levels, but that these impacts can be managed below this warming threshold (IPCC, 2018). Coupled with rising mean global temperature is a projected increase in the frequency, intensity, and duration of extreme heatwave events that have the potential to cripple crop yields (Battisti and Naylor, 2009; Perkins *et al.*, 2012; Hatfield and Prueger, 2015; Hoegh-Guldberg *et al.*, 2018). Additionally, some cropping areas, such as temperate, high-latitude regions, will likely face even greater warming than tropical regions of the world (Hoegh-Guldberg *et al.*, 2018). Therefore, there is an urgent need, first and foremost, for mitigation strategies to reduce fossil fuel emissions to cap warming at 1.5 °C (IPCC, 2018), but also for development of our major cropping systems to be more resilient to hotter growing seasons and extreme temperature events that seem inevitable in the coming century.

Global yield losses in key crops, such as maize and wheat, have been attributed to higher growing season temperatures (Lobell *et al.*, 2011; Lobell and Gourdji, 2012; Asseng *et al.*, 2015). Without crop improvement strategies, including genetic engineering and adaptation under carbon dioxide (CO_2_) fertilization, substantial yield declines per °C of warming have been projected for the major cropping systems of maize (7.4%), wheat (6.0%), rice (3.2%), and soybean (3.1%) (C. Zhao *et al.*, 2017). Yet, to keep pace with supplying food and fuel to the growing human population, agricultural production will need to double (based on average yield in 2005) over this century to meet increased caloric demand (Long and Ort, 2010; Ray *et al.*, 2013). Additionally, the full theoretical extent of the CO_2_ fertilization effect is unlikely to be realized due to the impact of rising temperature (Long *et al.*, 2006*a*; Ainsworth and Long, 2020). Thus, improving crop resilience to temperature stress is a vital step towards ensuring global food and fuel demands are met.

Temperature is a critical meteorological determinant of crop development and function. Temperature alters enzyme function within a leaf (Bernacchi *et al.*, 2001; Walker *et al.*, 2013; Florian *et al.*, 2014; Kumarathunge *et al.*, 2019; Timm *et al.*, 2019) and triggers changes in developmental growth stage that are tightly coupled with crop yield (Ruiz-Vera *et al.*, 2018; Zhu *et al.*, 2018). Furthermore, the amount of water vapour in air at saturation increases exponentially with temperature, raising the vapour pressure deficit (VPD), and driving more potential water loss from plants (Novick *et al.*, 2016; Grossiord *et al.*, 2020). The result of these broad crop physiological responses to temperature means that any shifts in long-term mean annual temperature and extreme temperature events will be likely to have significant impacts on crop production from the key food and fuel growing regions of the world.

Improvements in how crops function from the enzyme to ecosystem scale are required to maintain historic increases in crop yields into the future, whilst ensuring cropping systems remain resilient to rising temperatures (Long and Ort, 2010). Engineering improvements to photosynthesis, including its resilience to perform under hotter temperatures at the leaf, plant, and canopy levels, is an emergent strategy that may help boost yields (Long *et al.*, 2006*b*; Ainsworth and Ort, 2010; Ort *et al.*, 2015; Betti *et al.*, 2016; Kromdijk and Long, 2016; Kubis and Bar-Even, 2019; Posch *et al.*, 2019; Simkin *et al.*, 2019; Wu *et al.*, 2019; Furbank *et al.*, 2020). Developing better warning systems, such as early detection of crop ecosystem stress, will also improve targeted management approaches that reduce resource use (i.e. water and pesticides), expenditure, and time (Guanter *et al.*, 2014; Chlingaryan *et al.*, 2018; Camino *et al.*, 2019).

Realizing the full impact of temperature increase on crop photosynthesis across scales is an area of ongoing investigation, particularly given the complex interactions of water availability, increasing atmospheric CO_2_ concentrations ([CO_2_]), nutrient availability, and the increased frequency and/or intensity of extreme climate events that feed back to alter annual crop photosynthesis and productivity. There have been several seminal reviews on the effect of rising temperature on crop photosynthetic performance (Ainsworth and Ort, 2010), photosynthetic enzyme function (Slattery and Ort, 2019), plant carbon metabolism (Dusenge *et al.*, 2019), and plant development (Wang *et al.*, 2012), as well as global assessments of how crop yield is likely to change as temperatures rise (Lobell and Gourdji, 2012; C. Zhao *et al.*, 2017). Yet, reviews that address all these scales in one are limited.

**Fig. 1.**
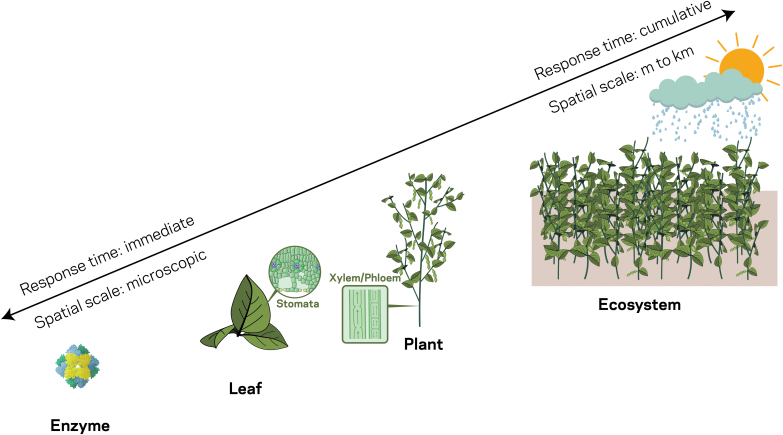
The spatial scale and temporal response time of photosynthetic processes in cropping systems from the enzyme to ecosystem scale.

This review focuses on synthesizing current advances in understanding the effects of temperature on cropping systems from the enzyme to ecosystem scale (Fig. 1) to provide a comprehensive assessment of how crop photosynthesis changes as temperature increases. Beginning at the enzyme scale, we discuss (i) within-leaf responses to temperature, followed by (ii) stomata and plant transport system responses to heat; (iii) temperature effects on whole plants and their development; and (iv) how each of these factors scales to the crop ecosystem to impact photosynthesis and annual yield (Fig. 1). Key abbreviations used throughout the review are listed and expanded in Table 1. For each scale discussed, we identify areas for research development that are needed to ensure the major crops that feed and fuel the world are more resilient to the impacts of rising temperature that will occur without implementation of climate mitigation strategies.

**Table 1.** Nomenclature and explanation of terms used across different scales

Abbreviation	Long name	Description
[CO_2_]	CO_2_ concentration	The concentration of carbon dioxide in the atmosphere, or within the leaf if specified as such
*A*	Assimilation	Net carbon assimilation during photosynthesis
E_a_	Activation energy	The input energy required to result in a chemical reaction
ER	Ecosystem respiration	Carbon consumed in an ecosystem by plants (autotrophic) or animals/microbes/fungi (heterotrophic)
ET	Evapotranspiration	Water loss through the processes of evaporation from surfaces and transpiration from leaves
FACE	Free air CO_2_ enrichment	An open-air experimental design that raises atmospheric [CO_2_] above ambient conditions experienced by plants at the ecosystem scale
FSPM	Functional and structural plant modelling	Models developed to simulate morphology and growth of single plants as they interact with their environment.
GPP	Gross primary productivity	Photosynthesis of all leaves and other photosynthetic plant parts represented at the ecosystem scale
g_s_	Stomatal conductance	A measure of the capacity for gaseous exchange of CO_2_ entering and water vapour leaving a leaf, measured as a molar flux on an area basis (mol m^–2^ s^–1^)
NEE	Net ecosystem exchange	A measure of the net flux of carbon between the land surface and the atmosphere
NSCs	Non-structural carbohydrates	Soluble sugars and starch that provide energy for plant growth and metabolism
PSII	Photosystem II	The first link in the electron transport chain of photosynthesis
QTLs	Quantitative trail loci	Sections of DNA (loci) that relate to a quantitative trait in the phenotype of an organism
Rca	Rubisco activase	An accessory protein that activates Rubisco
RA	Autotrophic respiration	Carbon consumed in an ecosystem by plants for growth and maintenance
RH	Heterotrophic respiration	Carbon consumed in an ecosystem by non-photosynthetic organisms
Rubisco	Ribulose-1,5-bisphosphate carboxylase/ oxygenase	Enzyme that all plants use to fix carbon dioxide as an entry point to the photosynthetic carbon reduction cycle. Rubisco also catalyses a reaction with oxygen, which is the first step in photorespiration
RuBP	Ribulose-1,5-bisphosphate	Five-carbon molecule that is used, along with CO_2_, as a substrate in photosynthesis in a reaction catalysed by Rubisco. RuBP will also bind with oxygen to initiate the process of photorespiration, also catalysed by Rubisco.
S_c/o_	Rubisco specificity	The specificity of Rubisco for binding CO_2_ compared with O_2_
SD	Stomatal density	The number of stomata per unit of leaf area
SIF	Sun-induced chlorophyll fluorescence	The emission of red light by plants during the process of sunlit photosynthesis
*T*_opt_	Thermal optimum	Describes an optimal temperature for driving a particular process
VPD	Vapour pressure deficit	A measure of the difference between the amount of moisture in the air and how much moisture air can hold before it becomes saturated.

## Temperature response of photosynthesis within the leaf: the critical role of enzyme function

**Fig. 2.**
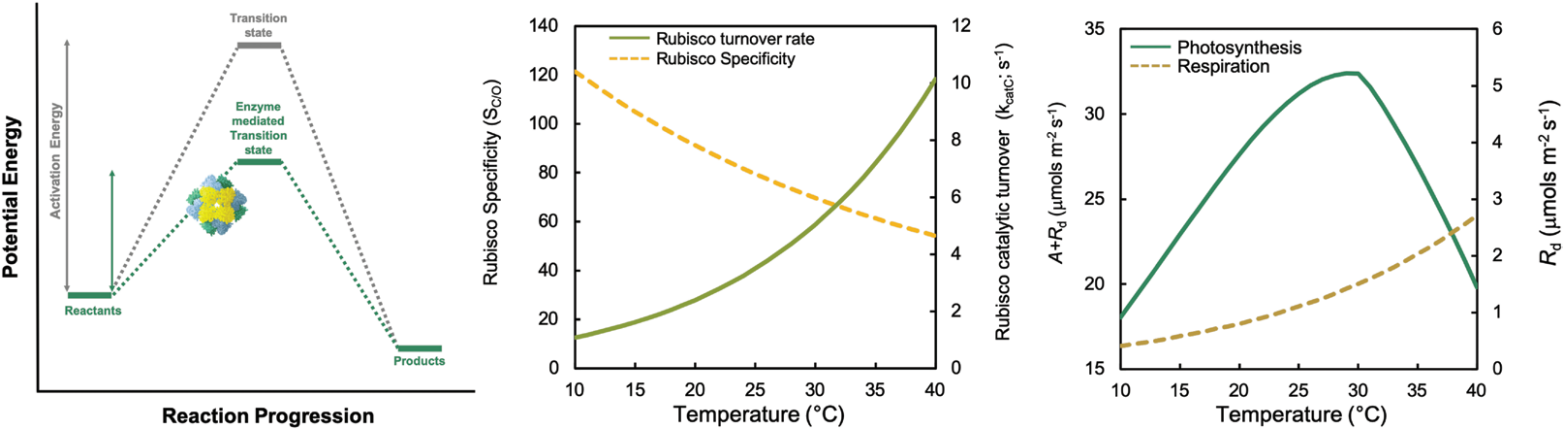
Temperature effects on enzyme-driven processes of photosynthesis. (A) Schematic energy profile of an exergonic chemical reaction. Enzymes, such as Rubisco, facilitate biochemical reaction progression by lowering the activation energy requirements of the transition state between reactants and product formation, though in the case of Rubisco this is simplified as the enzyme facilitates a multistep catalysis (Flamholz *et al.*, 2019). (B) Modelled temperature responses of tobacco Rubisco carboxylation catalytic turnover rate (green solid) and specificity for CO_2_ over O_2_ (yellow dashed line), using parameters from Orr *et al.* (2016) and temperature responses from Bernacchi *et al.* (2001). (C) Temperature response of gross photosynthesis (carbon assimilation *A*+mitochondrial respiration *R*_d_, green solid line) and of mitochondrial respiration (*R*_d_, gold dotted line) for an idealized C_3_ species. Data were modelled using the leaf model of photosynthesis (Farquhar *et al.*, 1980) with temperature adjustments (Bernacchi *et al.*, 2001).

Temperature regulation is foundational in biological systems, as chemical reaction rates are a function of the tissue temperature and the energy required to initiate the reaction—the activation energy (E_a_) (Fig. 2A). Enzymes lower this E_a_ barrier, enhancing the rate of enzyme-catalysed reactions driving biological metabolism (Wolfenden and Snider, 2001). In theory, reaction rates are predicted to increase exponentially with temperature. In reality, most biological temperature responses increase exponentially with temperature until reaching a thermal optimum (*T*_opt_), after which rates decline due to enzyme deactivation and denaturation at increasingly high temperatures (Fig. 2B, C).

The photosynthetic machinery within a leaf is a logical place to begin when considering the effects of temperature on crop photosynthesis, as many component processes of photosynthetic metabolism are highly temperature sensitive. At a biochemical level, net photosynthetic carbon assimilation (*A*) is largely determined by Rubisco efficiency and activation, and ribulose bisphosphate (RuBP) regeneration (Table 1) (Farquhar *et al.*, 1980). The predominant determinant varies with chloroplastic [CO_2_]; RuBP regeneration limits *A* at elevated [CO_2_], but Rubisco performance limits *A* at ambient and subambient [CO_2_]. Enzyme degradation at elevated temperatures can impede the function of PSII, decrease electron transport rates, inhibit Rubisco activase (Rca), and decrease chlorophyll content (Salvucci *et al.*, 2001; Guo *et al.*, 2006; Allakhverdiev *et al.*, 2008; Prasad and Djanaguiraman, 2011). Elevated temperature can also induce membrane permeability, leading to direct damage of the chloroplast thylakoid membranes, which further inhibits light harvesting, electron transport rates, and ATP generation (Schrader *et al.*, 2004; Prasad *et al.*, 2008; Djanaguiraman *et al.*, 2013; Pokharel *et al.*, 2020). However, thermal lability of enzymes directly involved in *A* remains the major cause of photosynthetic inhibition of C_3_ and C_4_ crops grown under elevated temperatures (Crafts-Brandner and Salvucci, 2000; Schrader *et al.*, 2004; Sage and Kubien, 2007; Perdomo *et al.*, 2016; Slattery and Ort, 2019).

The optimal temperature of RuBP regeneration is generally higher than that of Rubisco carboxylation (Hikosaka *et al.*, 2006); therefore, under current atmospheric [CO_2_] and saturating light, the temperature dependence of photosynthesis is well explained by Rubisco biochemistry (Sage and Kubien, 2007). As temperatures increase, the fraction of enzyme able to meet or exceed the E_a_ required for catalysis increases, and so Rubisco carboxylation activity increases (Fig. 2B). However, Rubisco is a bi-functional enzyme, also catalysing the oxygenation of RuBP (Ogren and Bowes, 1971; Tcherkez, 2016; Bathellier *et al.*, 2020; von Caemmerer, 2020). The specificity of Rubisco for CO_2_ versus O_2_ (S_C/O_) declines as temperatures increase, decreasing the ratio of carboxylation to oxygenation *in vivo* (Fig. 2B). This increased propensity for Rubisco oxygenation at elevated temperatures produces more 2-phosphoglycolate, which must be cycled through the photorespiratory pathway, resulting in a loss of previously fixed carbon at an energetic expense (Walker *et al.*, 2016).

In C_4_ photosynthesis, CO_2_ is concentrated around Rubisco in bundle sheath chloroplasts. Thus, stimulation of photorespiration by elevated temperatures is minimal, and *A* in C_4_ plants has a higher *T*_opt_ than in C_3_ plants (Sage and Kubien, 2007). Above the *T*_opt_, C_4_ photosynthesis may also be limited through inactivation of Rubisco (Crafts-Brandner and Salvucci, 2002), or by rates of other C_4_ bundle sheath enzymes (Boyd *et al.*, 2015), which show species-specific temperature responses (Sonawane *et al.*, 2017). This impact is evident in field-grown maize, where leaf-level *A* and yield decline with elevated temperature, even under elevated CO_2_ conditions (Ruiz-Vera *et al.*, 2015).

The duration and intensity of future warming events are both projected to change (Hoegh-Guldberg *et al.*, 2018), resulting in significant impacts on any potential thermal acclimation of *A* (Kattge and Knorr, 2007; Vico *et al.*, 2019). In sunlit leaves near the top of the canopy, photosynthetic acclimation through increased electron transport capacity, differential expression of Rca isoforms, and heat shock protein expression can occur with long-term growth at warmer temperatures (Yamori *et al.*, 2014). However, short-term temperature increases can increase leaf respiration, resulting in lower *A* compared with those at ambient temperature, and a strong and relatively rapid acclimation response that reduces the effect as higher temperatures persist (Way and Yamori, 2014; Kumarathunge *et al.*, 2019). During heatwaves or acute heat stress, defined by sudden increases in temperature (Smith and Dukes, 2017) with significant but reversible effects on photosynthesis (Siebers *et al.*, 2015, 2017; Thomey *et al.*, 2019), the acclimation responses may be too slow or small to confer a measurable benefit. In these situations, energy balances will shift as rates of photosynthesis decline above the *T*_opt_ and respiration rates increase (Fig. 2C). Thus, most opportunities for improving crop productivity in a warmer world focus on improving photosynthetic carbon gain above *T*_opt_.

### Recent advances made at the leaf level to improve understanding on temperature effects

**Fig. 3.**
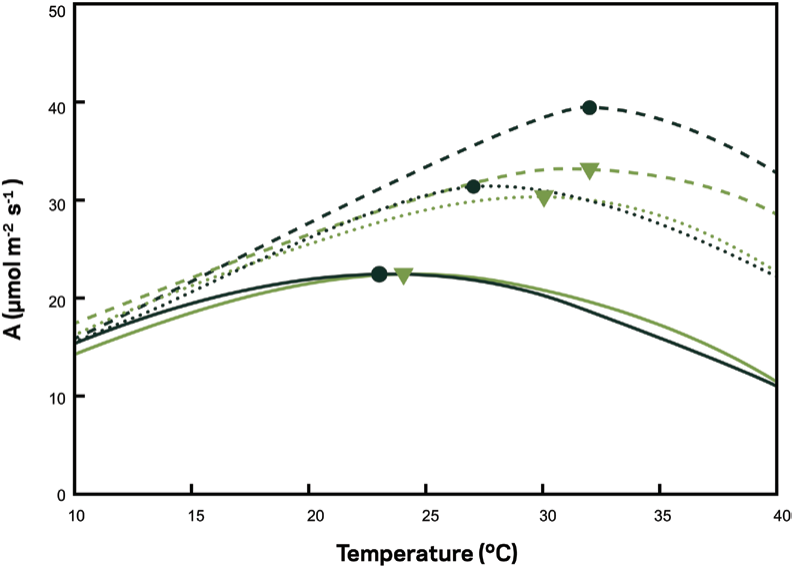
Temperature response of C_3_ leaf photosynthesis (μmol m^–2^ s^–1^) modelled at atmospheric [CO_2_] of 400 (solid lines), 600 (dotted lined), and 800 (dashed lines) μmol mol^-–1^. Model parameters were taken from Bernacchi *et al.* (2001, 2003, black circles) and Long (1991, green triangles), with the symbol location on the curve representing the temperature optimum for each photosynthetic response curve. The figure has been redrawn from Bagley *et al.* (2015), with permission.

The response of *A* to a wide range of environmental conditions is well understood based on the leaf model of photosynthesis (Farquhar *et al.*, 1980; Long, 1991). Despite the mechanistic understanding of modelled predictions, there remain significant uncertainties. For example, the leaf photosynthesis model (Farquhar *et al.*, 1980) was recently parameterized using values measured from C_3_ plants grown under field conditions exposed to supplemental heating (Bagley *et al.*, 2015). The results demonstrate that growth at higher temperatures does not translate to a higher *T*_opt_ but does lower photosynthetic rates at all temperatures. An interaction between warmer temperature and elevated [CO_2_] was observed; however, acclimation of photosynthetic enzymatic activity to higher temperature negatively impacted the benefit of higher CO_2_ (Fig. 3) (Bagley *et al.*, 2015). These results demonstrate the challenges associated with temperature, namely that short- and long-term responses of photosynthesis are complex and are complicated by other environmental variables.

Despite the complex interaction between temperature and photosynthesis, promising strategies have been identified to increase photosynthetic *A* at higher temperatures by either enhancing RuBP carboxylation or improving energy efficiency of photorespiration. The limitations imposed by Rubisco include a slow catalytic rate, competitive inhibition by O_2_, and activation requirement via heat-sensitive Rca. Strategies to improve our understanding of Rubisco are needed to overcome these temperature impacts.

Rubisco has long been a target for modification to improve its catalytic rate and substrate specificity (Somerville and Ogren, 1982; Zhu *et al.*, 2004; Sharwood, 2017). For example, an apparent trade-off between catalytic rate and specificity hinders progress for exploitation (Tcherkez *et al.*, 2006; Savir *et al.*, 2010; Flamholz *et al.*, 2019). Recently, a systematic survey of prokaryotic Rubisco has identified the fastest version of the enzyme measured to date (22 s^–1^), but it still displays characteristically poor substrate specificity (Davidi *et al.*, 2020). Screening for natural variation in Rubisco performance has uncovered kinetic diversity among land plants that would confer a predicted benefit to crop *A*, particularly at elevated temperatures (Galmés *et al.*, 2015; Orr *et al.*, 2016; Sharwood *et al.*, 2016). Unique combinations of Rubisco small and large subunits from different species also provide an opportunity to optimize kinetic performance at higher temperatures (Lin *et al.*, 2020; Martin-Avila *et al.*, 2020; Sakoda *et al.*, 2020). Finally, the newfound ability to assemble plant Rubisco in a bacterial host will enable both structure–function comparisons and directed evolution studies to identify novel mutations to improve Rubisco performance (Aigner *et al.*, 2017; Zhou and Whitney, 2019).

Rca regulates Rubisco activity by displacing inhibitory sugar phosphates from the catalytic site of Rubisco. Although Rubisco remains active up to 50 °C *in vitro*, Rca activity declines well below this temperature (Salvucci and Crafts-Brandner, 2004; Galmés *et al.*, 2016), and thus can limit photosynthesis at high temperatures. The production of inhibitory catalytic misfire products increases with temperature, implying that the role of Rca also becomes increasingly important. However, when measured *in vitro*, the rate of spontaneous release of these inhibitors also increases at elevated temperatures, resulting in less inhibition of Rubisco activity, which contradicts this assumption (Schrader *et al.*, 2006; Carmo-Silva *et al.*, 2015; Bracher *et al.*, 2017). Despite this, manipulating Rca thermostability has improved photosynthetic thermotolerance in Arabidopsis (Kurek *et al.*, 2007; Kumar *et al.*, 2009) and rice (Wang *et al.*, 2010; Scafaro *et al.*, 2016, 2018; Shivhare and Mueller-Cajar, 2017), motivating research efforts to enhance the thermotolerance of Rca in other crops. Exploiting temperature-induced differential expression of Rca is a potential strategy to accomplish this objective. In many crops, Rca consists of multiple protein isoforms with differing heat sensitivity (Crafts-Brandner *et al.*, 1997; Law *et al.*, 2001; Law and Crafts-Brandner, 2001; Carmo-Silva *et al.*, 2015; Scafaro *et al.*, 2019; Kim *et al.*, 2020). In bread wheat, altered thermal tolerance between Rca isoforms is conferred by a single amino acid substitution that acts as a thermal and regulatory switch, providing a compelling target for future genome editing efforts (Scafaro *et al.*, 2019; Degen *et al.*, 2020).

The photorespiratory pathway recycles the inhibitory by-products of Rubisco oxygenation, which releases previously fixed carbon and ammonium that is energetically costly to re-fix. Photorespiratory CO_2_ loss limits productivity in C_3_ plants, reducing crop yields by >20% in soy and wheat (Walker *et al.*, 2016). Engineering carbon-concentrating mechanisms (CCMs) to directly increase the [CO_2_] at the site of Rubisco represents one strategy for stimulating carboxylation over oxygenation (Long *et al.*, 2018; Atkinson *et al.*, 2020). This can be accomplished via the introduction of a biophysical CCM, such as those found in cyanobacteria and algae (Hennacy and Jonikas, 2020), or via the conversion of C_3_ photosynthesis to C_4_ or C_2_ types. Researchers have recently established a functioning C_4_ pathway in rice by transformation with a single construct harbouring coding sequences for five enzymes, although expression will require optimization before any benefit is realized (Ermakova *et al.*, 2020). Engineering C_2_ photosynthesis, a simple CCM that captures, concentrates, and re-assimilates photorespired CO_2_, is a promising approach currently in its infancy. An advantage of C_2_ photosynthesis is the ability to exploit native genes and alter only their regulation and expression, as all required genes are present in C_3_ species (Lundgren, 2020). Finally, direct manipulations of the photorespiratory pathway can lower the cost of photorespiration. Overexpression of native photorespiratory genes can enhance *A* and growth, probably altering the balance between photosynthesis and photorespiration (Timm *et al.*, 2012, 2015, 2018; Flügel *et al.*, 2017; López-Calcagno *et al.*, 2019). Synthetic glycolate metabolic pathways using enzymes from other organisms in combination with RNAi to limit glycolate flux through the native pathway increase tobacco biomass under field-grown conditions (South *et al.*, 2019). Similarly, an alternative photorespiratory pathway introduced into rice using three rice enzymes improved *A*, leading to increased aboveground biomass, but displayed inconsistent improvements in yield (Shen *et al.*, 2019). Further carbon-conserving glycolate metabolic pathways have also been designed and tested *in vitro* (Trudeau *et al.*, 2018; Ross *et al.*, 2020). While these and the previous strategies to enhance photosynthetic performance above the *T*_opt_ hold potential to improve crop performance, testing in food and fuel crops over diverse environmental ranges will provide the key validation of their efficacy.

## Temperature impacts on stomata and plant transport systems

Scaling the response of plant photosynthesis, from the chloroplast to leaf or whole plant, involves CO_2_ diffusion to the site of the chloroplast, as well as subsequent photosynthate transport throughout the plant. To reach the site of carboxylation within chloroplasts, CO_2_ must first diffuse from the atmosphere to the substomatal cavities, then through the intercellular airspaces to the chloroplast. This gaseous diffusion imposes a restriction on CO_2_ availability in the chloroplast that depends on the CO_2_ conductance through the leaf boundary layer, stomata, and intercellular environment (i.e. mesophyll conductance). The temperature response of mesophyll conductance varies between species, and can impose a limitation on carbon fixation, which has been well reviewed (Niinemets *et al.*, 2009; Flexas *et al.*, 2012, 2014; von Caemmerer and Evans, 2015). In this section, we discuss temperature impacts on stomata, as well as the plant transport systems that move photosynthate from leaves to other parts of the plant for growth, maintenance, and storage.

### How stomatal function links leaf to whole-plant photosynthesis

Stomata control the majority of gaseous exchange between the atmosphere and the leaf interior. Therefore, stomatal behaviour is critically important for CO_2_ uptake to meet photosynthetic demand and for controlling leaf water loss that impacts evaporative cooling, nutrient uptake, and plant water status (Lawson *et al.*, 2010; Matthews and Lawson, 2019; Lawson and Matthews, 2020). Stomata open and close in response to various environmental signals and internal leaf conditions. In general, conditions of high or increasing light intensity, low (internal) [CO_2_], and low VPD open stomata, whilst closure is observed under opposite conditions (Matthews and Lawson, 2019). Stomatal conductance (*g*_s_) provides a measure of the capacity for gaseous exchange of water vapour leaving the leaf (Table 1), and is determined by the number of stomata per unit leaf area and the size of the pore aperture. Thus, alterations in both leaf morphological features and leaf functional responses to external meteorological forcing can influence *g*_s_, which in turn can impact photosynthesis and overall crop performance.

According to the optimization hypothesis, plants coordinate *g*_s_ and *A* to maximize *A* whilst minimizing water loss (Cowan and Farquhar, 1977; Lawson *et al.*, 2010; Buckley *et al.*, 2017). However, this is not always the case, as a decoupling between *g*_s_ and *A* has been reported (von Caemmerer and Evans, 2015; Urban *et al.*, 2017), whereby stomata open to increase leaf cooling despite the suppression of *A* (Drake *et al.*, 2018). A positive correlation between steady-state *g*_s_ and yield has been observed in the field (Fischer *et al.*, 1998; Fischer and Rebetzke, 2018), reflecting the control stomata exert on CO_2_ uptake for photosynthesis and on evaporative cooling. Temperature can severely limit stomatal performance and consequently yield, especially in temperature-sensitive crops such as wheat, where evaporative cooling to maintain *T*_opt_ can be more important than removal of diffusional constraints for photosynthesis (Fischer *et al.*, 1998; Lu *et al.*, 1998). The same environmental cues that stimulate changes in stomatal aperture can also induce alterations to the stomatal density (SD) per unit leaf area and their distribution across the leaf (Weyers *et al.*, 1997; Weyers and Lawson, 1997), which impacts *g*_s_ with implications for *A*. Changes in one anatomical trait (i.e. SD) are often compensated for by modifications in another (i.e. stomatal size), with many studies reporting a strong negative correlation between SD and size (e.g. Drake *et al.*, 2013). However, while this relationship appears in closely related species (Faralli *et al.*, 2019), it does not hold across multiple diverse species (McAusland *et al.*, 2016).

One of the most well-studied impacts of environment on stomatal numbers is atmospheric [CO_2_], which has been demonstrated to decrease SD with increasing [CO_2_] in a number of different species (Hetherington and Woodward, 2003), including several major cropping systems (Ainsworth and Rogers, 2007). Global warming associated with rising [CO_2_] has been shown to increase SD in several crop species (Rodrigues *et al.*, 2016; Caine *et al.*, 2019) including soybean (Jumrani *et al.*, 2017), tobacco (Hu *et al.*, 2014), and grape (Rogiers *et al.*, 2011), often with concurrent decreases in stomatal size (Rodrigues *et al.*, 2016), although no effect was reported for maize (Zheng *et al.*, 2013). However, such changes in anatomy (i.e. SD or guard cell length) do not necessarily translate into differences in *g*_s_, and vice versa (Rodrigues *et al.*, 2016; Kapadiya *et al.*, 2017), illustrating the importance of considering both functional responses and anatomical alterations with growth temperature.

### Stomatal behavioural responses to elevated temperature

Whilst higher temperatures can disrupt a number of metabolic processes, including those that take place in the guard cells, stomatal response to high temperatures is often complicated by the fact that temperature also affects photosynthesis, VPD, transpiration, and plant water status, which all feed back on stomatal behaviour (Urban *et al.*, 2017). Changes in temperature alter VPD (see Scaling from plants to ecosystem), which subsequently alters transpiration as stomata respond to the change in atmospheric dryness (e.g. Brodribb and McAdam, 2011; Merilo *et al.*, 2018). Higher VPD increases the leaf–atmosphere diffusion gradient, driving greater water loss and triggering stomatal closure to maintain plant water status (Mott and Peak, 2013). The actual mechanisms for stomatal response to VPD are still not fully elucidated, except for a broad classification into two hydraulic responses: active and passive (Xie *et al.*, 2006; Chater *et al.*, 2011; Bauer *et al.*, 2013; Franks, 2013; McAdam and Brodribb, 2014).

**Fig. 4.**
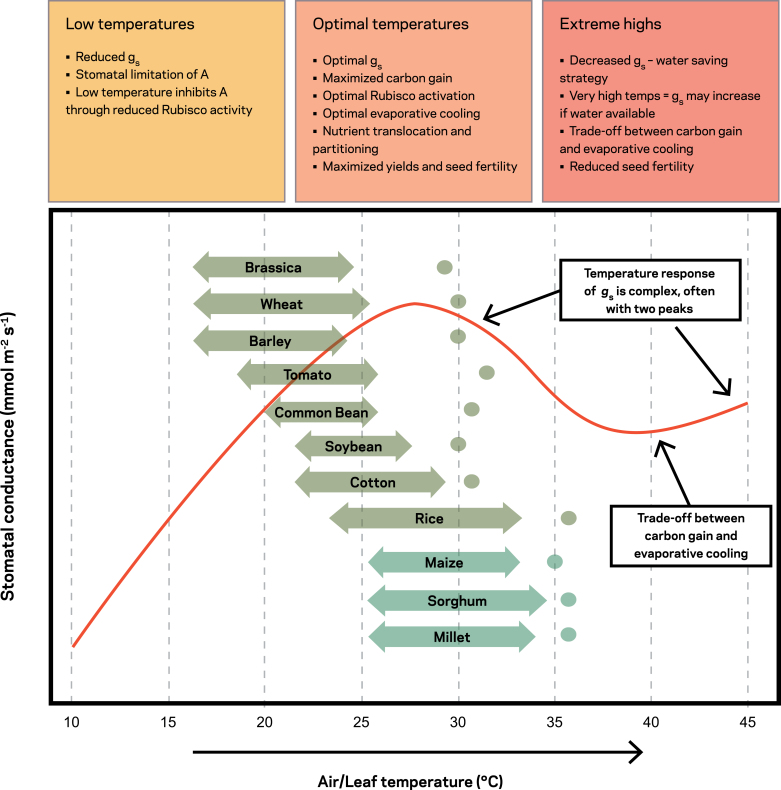
Impact of temperature on changes in stomatal conductance and response in major cropping systems. Highlighted is a generic response of stomatal conductance (*g*_s_) across a temperature range (red line); optimal temperature ranges for major global crop types (two-headed arrows), including critical temperatures when biomass and yield are significantly reduced (dots). Reproduced with permission from Matthews and Lawson (2019).

Studies examining stomatal responses specifically to temperature have received less attention than those focusing on other environmental factors (Way, 2011; Teskey *et al.*, 2015), and the findings are highly variable between species (Sage and Kubien, 2007; Matthews and Lawson, 2019). *g*_s_ has a mixed response with rising temperature across crop species (Schulze *et al.*, 1975; Lu *et al.*, 2000; von Caemmerer and Evans, 2015; Urban *et al.*, 2017), with an increase in *g*_s_ of 163% observed in maize (Zheng *et al.*, 2013), yet a decrease (Sage and Sharkey, 1987; Raven *et al.*, 2005) or no effect on *g*_s_ at all with increased temperature reported in other crops (Sage and Sharkey, 1987; Aphalo and Jarvis, 1991; von Caemmerer and Evans, 2015). Generalizing stomatal response to changes in leaf temperature is complicated by interactions between temperature and VPD, but also by the non-linearity in responses, often described as bell shaped (Fig. 4) (Way, 2011; Matthews and Lawson, 2019). *g*_s_ tends to increase with temperature up to a tipping point (Way, 2011; Tricker *et al.*, 2018) before rapidly decreasing at greater temperatures (Šantrůcek and Sage, 1996), and can increase again if stomata reopen at very high temperatures (Fig. 4). The temperature where stomata commence closure in the bell-shaped response is species specific and dependent on the growth temperature conditions (Sage and Sharkey, 1987). It is likely that this variation can be explained by differences in hydraulic conductance and temperature effects on viscosity (Cochard *et al.*, 2000), as well as photosynthetic demand (Šantrůcek and Sage, 1996).

Heat stress induces responses in *g*_s_ that vary genotypically (Zhou *et al.*, 2017; Ferguson *et al.*, 2020); however, whether this variation in *g*_s_ can be linked to heat sensitivity levels remains unclear. Plants can also acclimate to different growth temperatures, resulting in lower stomatal sensitivity to short-term (i.e. minutes) changes in ambient temperature (e.g. Šantrůcek and Sage, 1996). Under different growth temperatures, the *g*_s_ response that plants exhibit can be a similar shape, though the magnitude can vary greatly (Yamori *et al.*, 2006; Way, 2011).

Increased *g*_s_ values at higher temperatures will benefit plant performance by removing diffusional constraints on CO_2_ diffusion into the leaf, and the resulting increase in intercellular CO_2_ will help to reduce the negative impact of increased photorespiration at higher leaf temperatures (see previous section). Additionally, higher *g*_s_ will facilitate enhanced transpiration and evaporative cooling, which will support the maintenance of leaf temperature closer to the *T*_opt_ for photosynthesis, further reducing photorespiratory processes (Urban *et al.*, 2017). However, the increased water loss through higher *g*_s_ can compromise plant water status (Matthews and Lawson, 2019) which, depending on the degree of water stress, could be detrimental to plant performance and growth. Furthermore, high atmospheric temperatures often occur in conjunction with reduced water availability, so stomatal temperature responses are linked closely not only with VPD but also with drought and water potential (Urban *et al.*, 2017). Stomata close when water becomes limiting to avoid catastrophic water loss, even when demands for photosynthesis are high, demonstrating the hierarchal response of one signal over-riding others. As *g*_s_ decreases with rising temperature and/or limited water availability, leaf temperature will further increase due to reduced evaporative cooling, leading to metabolic disruptions (Tezara *et al.*, 1999; Perdomo *et al.*, 2017), and lower photosynthesis from restricted CO_2_ diffusion (Chaves *et al.*, 2003).

### Advancements needed to improve stomatal resilience to heat stress

Manipulation of stomatal anatomy and metabolism has been suggested as a potential mechanism for crop improvement under adverse environmental conditions. SD has been altered via manipulating the stomatal development pathway, which can be achieved by focusing on the epidermal patterning factor family of transcription factors (EPFs). Many studies suggest that decreasing SD will reduce water loss and improve water use efficiency (Hughes *et al.*, 2017; Caine *et al.*, 2019), but this could also increase leaf temperatures. However, rice with reduced SD (due to increased expression of osEPF1) showed reduced water use that resulted in lower leaf temperature relative to wild-type controls under drought stress (Caine *et al.*, 2019). Conversely, overexpression of *EPF9/Stomagen* results in increased *g*_s_ and *A*, but at the expense of water use efficiency (Tanaka *et al.*, 2013). Masle *et al.* (2005) demonstrated in Arabidopsis that the ERECTA gene not only influenced SD (and subsequently *g*_s_), but also the coordination between *A* and *g*_s_, which offers the potential to manipulate transpiration efficiency. Thus, it would be interesting to explore the potential of these mutants under different water, temperature, and VPD stress conditions (Lawson *et al.*, 2014).

Manipulating guard cell metabolism or signalling pathways is an alternative and mostly unexplored avenue for future consideration (Lawson and Blatt, 2014; Lawson *et al.*, 2014). For example, Hettenhausen *et al.* (2012) manipulated a mitogen-activated protein kinase, MPK4, in tobacco that results in increased *g*_s_, whilst overexpression of aquaporins in rice and grapevine increases *g*_s_ and *A* under both stress and non-stress conditions (Hanba *et al.*, 2004; Sade *et al.*, 2010). There are many other examples where components of guard cell osmoregulation and/or mesophyll metabolism have altered stomatal function (see table 1 in Matthews and Lawson, 2019) that provide a mostly unexploited genetic reservoir of material to explore for manipulating stomatal behaviour to cope with global warming. Altogether, these studies suggest that manipulation of stomatal anatomy and function could be a promising path to increase evaporative cooling as a strategy to cope with future climate conditions, but this may increase water requirements as a consequence.

The detrimental effect of elevated temperature is often associated with impacts on leaf biochemistry; however, for some crops, the main cause of decreased yield is due to high temperature during the reproductive stage of growth (Akter and Islam, 2017). Therefore, manipulating SD and stomatal function in non-foliar tissue may also be an important and overlooked route for reducing temperature stress at key times (Simkin *et al.*, 2020). Furthermore, the function of stomata in both foliar and non-foliar tissue and the role they play in translocation of photosynthate from source to sink tissues, including grain yield, is often ignored, as bulk flow within the phloem requires bulk flow of water in the xylem, which is a direct result of transpirational water loss that is ultimately controlled by stomata. Additionally, coordination between SD and minor vein density, which is a principle determinant of leaf hydraulic capacity (Brodribb *et al.*, 2007), has been observed in many species contributing to the balance between leaf water supply and demand (W.-L. Zhao *et al.*, 2017). The effect of rising temperature on this relationship requires further investigation, since trends differ across species (Hu *et al.*, 2014; Yang *et al.*, 2020).

### Temperature impacts on source to sink allocation and phloem transport

Carbohydrate translocation from photosynthetic source tissues (sources) to non-photosynthetic sink tissues (sinks) via the phloem is critical for vegetative and reproductive development, and ultimately crop yield. Alterations in plant source–sink balances, often induced by environmental stress such as high temperature, can impair carbohydrate allocation and negatively impact photosynthetic capacity and yield. Generally, heat stress decreases photosynthetic efficiency while increasing respiration and photorespiration rates (see earlier) and can affect reproductive development (Prasad *et al.*, 2017; Ferguson *et al.*, 2021), which shifts the dynamics between sources and sinks. Thus, a better understanding of these mechanisms is crucial to maintain crop productivity in a warmer world.

Alongside reduced photosynthesis, declines in leaf non-structural carbohydrate (NSC) contents have been reported in several crop species (including soybean, chickpea, castor bean, and maize) with short-term (≤7 d) exposure to heat stress (Kaushal *et al.*, 2013; Ribeiro *et al.*, 2014; Sun *et al.*, 2016; Thomey *et al.*, 2019). In tomato, maintained or higher levels of NSC in mature leaves were associated with heat tolerance under short-term heat stress (Zhou *et al.*, 2017), which could help fuel increased respiration (Ferguson *et al.*, 2021). However, under longer term heat stress, NSC accumulation in leaves and stems (tomato and rice, Zhang *et al.*, 2012; Zhang *et al.*, 2018) decreases root to shoot biomass ratio (castor bean, Ribeiro *et al.*, 2014), and the reduced carbon export rate from leaves suggests a reduction in carbohydrate export towards sinks (maize, Suwa *et al.*, 2010). Carbohydrate accumulation in mesophyll cells has been linked to down-regulation of photosynthetic capacity via negative feedback on Rubisco content and activity (Moore *et al.*, 1999; Long *et al.*, 2004). Yet any potential regulatory role for leaf carbohydrate accumulation observed during long-term heat stress remains unclear, due to the direct impact of temperature on Rubisco (see earlier).

**Fig. 5.**
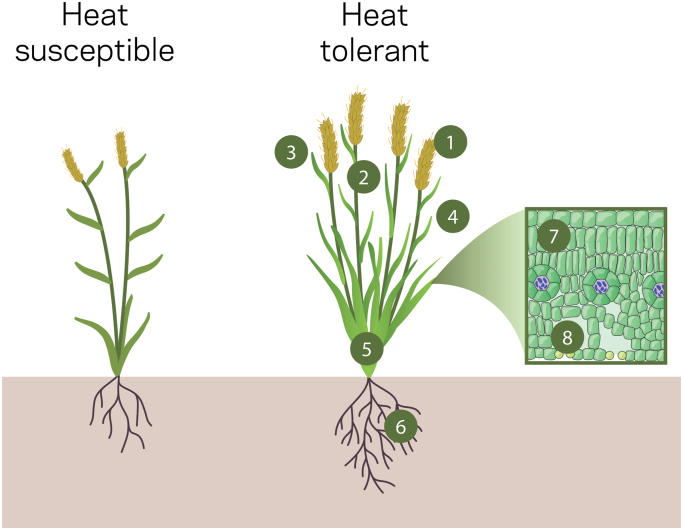
Structural and functional attributes that make a crop plant more susceptible (left) or tolerant (right) to heat stress. Numbers indicate the following: (1) higher invertase activity in spike/grain to maintain or increase carbohydrate import; (ii) remobilization of non-structural carbohydrates from the stems towards the spike/grain; (iii) short/erect flag leaf avoids direct light penetration and scorching, and has higher sucrose transporter expression to help maintain phloem loading and carbohydrate allocation to non-photosynthetic tissues; (iv) short/erect leaves avoid direct heat exposure, with angled leaves allowing light penetration lower into the canopy to help keep all leaves closer to temperature optimum; waxy leaves also help reduce water loss; (5) extra tillers and leaves to help maintain green leaf area and delay senescence; (6) more roots that reach deeper to access more soil moisture; (7) concentrated chlorophyll in the ‘sweet spot’ (i.e. not all in the top leaves) to improve leaf temperature optima; and (8) increased leaf stomatal density to improve CO_2_ entry into the leaves.

Remobilization of NSCs stored in intermediate sinks, such as stems, contributes to grain allocation especially in cereal crops, and could help compensate for reduced *A* when heat stress occurs at certain development stages (Fig. 5) (Blum *et al.*, 1994; Morita and Nakano, 2011; Zamani *et al.*, 2014; Xu *et al.*, 2020; Zhen *et al.*, 2020; Ferguson *et al.*, 2021). However, heat stress can also reduce stem NSC translocation efficiency decreasing yield further (Zamani *et al.*, 2014; Zhen *et al.*, 2020). Together, these studies suggest a negative impact of heat stress on carbohydrate translocation, especially towards the reproductive sinks, which highlights the importance of maintaining these functions to preserve yield in resilient crop cultivars.

Various modifications in phloem structure and function, which may affect carbohydrate transport and allocation in response to elevated temperature and heat stress, have been described in several crop species (Fig. 5). At a biochemical level, intraspecific variation in rice shows that maintained or increased expression of sucrose transporters in leaves, stems, and grains is related to heat tolerance (Miyazaki *et al.*, 2013; Phan *et al.*, 2013; Zhang *et al.*, 2018; Yaliang *et al.*, 2020), particularly for transporters thought to be involved in phloem loading and apoplastic sucrose retrieval along the transport pathway (Scofield *et al.*, 2007; Julius *et al.*, 2017). These findings suggest that sucrose transporters are promising targets to develop heat-resilient crop cultivars. Invertases and sucrose synthases may also be interesting targets for crop improvement under heat stress (Julius *et al.*, 2017; Xu *et al.*, 2020). By catalysing sucrose degradation in sinks, they increase the amount of sucrose being unloaded from the phloem into these sinks. Increased or maintained expression and/or activity of invertases and sucrose synthases in reproductive sinks has been linked to heat tolerance in several crop species including rice, tomato, and chickpea (Pressman *et al.*, 2006; Li *et al.*, 2012; Kaushal *et al.*, 2013; Phan *et al.*, 2013; Li *et al.*, 2015; Bahuguna *et al.*, 2017; Rezaul *et al.*, 2019; Yaliang *et al.*, 2020). With photosynthetic improvements to heat stress, the enzymes involved in sucrose transport and metabolism may become increasingly important for ensuring increased photosynthates reach vegetative and reproductive sinks.

At a structural level, deposition of callose (a polysaccharide) and protein conformational change were observed in broad bean phloem following heat shock, resulting in blocked phloem transport (Furch *et al.*, 2007). Heat-triggered callose deposition was also found in rice leaf and sheath plasmodesmata, especially in a heat-sensitive mutant with impaired carbohydrate translocation, potentially blocking phloem loading and/or unloading (Zhang *et al.*, 2018). The underlying mechanisms of callose deposition in phloem under heat stress still need further investigation. Additionally, phloem anatomical features, such as the number and cross-sectional area of phloem cells, are correlated with photosynthetic capacity and environmental conditions (Cohu *et al.*, 2014; Muller *et al.*, 2014; Adams *et al.*, 2016; Stewart *et al.*, 2016). Elevated temperature decreased phloem cell number and area in an Arabidopsis ecotype from a cool climate, correlating with reduced photosynthetic capacity compared with growth at lower temperature (Adams *et al.*, 2016; Stewart *et al.*, 2016). This highlights the need for comparative studies in major food and fuel crops to inform acclimation potential to elevated temperatures, and identify anatomical features to select for future crop varieties.

## Adding complexity: leaf interactions influence whole-plant responses to temperature

Scaling from enzymes functioning within a single leaf to a collective of leaves that make up a single plant adds a layer of complexity to the relationship between temperature and photosynthesis. The interaction of individual leaves within and among plants modifies the microclimate or phylloclimate (Chelle, 2005), causing variation in individual leaf temperatures within a crop plant. Leaf temperature depends on the leaf energy balance, including radiation, convection, and transpiration processes (Jones, 1993; Lambers *et al.*, 1998). Shading of lower leaves by leaves higher in the canopy drives exponential declines in light availability in crop canopies (Monteith, 1965), while leaves and stems present physical barriers to wind, reducing wind speed with canopy depth (Jacobs *et al.*, 1995). Air temperature, VPD, and [CO_2_] profiles influence gas exchange between the plant and the atmosphere. Thus, the interactions among all of these variables influence leaf temperature profiles with canopy depth.

Improving whole-plant photosynthesis has focused on the plant ‘ideotype’ that best intercepts light for optimal photon capture and utilization by light-harvesting complexes (Long *et al.*, 2006*b*; Ort and Melis, 2011). While temperature effects are usually secondary to optimal photon capture, work to improve light distribution within plant canopies may alleviate some of the limitations posed by plant temperature gradients (Fig. 5). Modelling suggests that less light absorption by upper canopy leaves could result in cooler leaf temperatures at the top of the plant (Drewry *et al.*, 2014), allowing those leaves to operate nearer *T*_opt_, which would be especially beneficial under heat stress conditions when *g*_s_ is limited. Shifting a greater proportion of photosynthesis to the lower canopy where wind speeds are lower and humidity is higher could also increase water use efficiency (Drewry *et al.*, 2014). However, the effects on leaf temperature remain uncertain.

### How a crop plant develops under heat stress and what this means for photosynthesis and yield

While leaf temperatures higher than *T*_opt_ directly affect whole-plant photosynthesis, they also have indirect impacts at plant and canopy scales across all stages of a plant’s life cycle. During the vegetative stage, deviation from a *T*_opt_ alters plant development and subsequently limits *A* for biomass accumulation. Heat stress reduces germination, seedling vigour, and establishment in soybean and cowpea (Covell *et al.*, 1986), and radicle elongation in rice (Han *et al.*, 2009). In maize, extreme heat reduces, and can completely halt, coleoptile growth (Weaich *et al.*, 1996). After plant establishment, heat stress can prevent leaf development (i.e. cassava, Burns *et al.*, 2010), thereby preventing leaf area accumulation for photosynthetic gain to the plant canopy (Fig. 5). For example, daytime temperatures >33 °C and high night-time temperatures reduce leaf emergence and tillering in rice, thereby reducing plant biomass (Chaudhary and Ghildyal, 1970; Fahad *et al.*, 2016*a*).

Heat damage to leaf photosynthetic pigments reduces photosynthetic efficiency during vegetative growth, which impacts biomass accumulation and development to reduce crop yield. For example, temperatures >35 °C negatively impact maize biomass accumulation due to degradation of chlorophyll, consequently reducing photosynthetic light absorption (Hatfield *et al.*, 2011; Hussain *et al.*, 2019). Premature loss of leaf chlorophyll due to heat stress accelerates mobilization of photosynthate to newer leaves and triggers early maturity of the whole plant (Nooden, 1986). This drives a shorter plant life cycle and reduces the grain-filling window—a critical yield determinant period for cereal plants. Heat-induced reductions in life cycle length have caused grain yield reduction in wheat (Camp *et al.*, 1982; Nicolas *et al.*, 1984; Reynolds *et al.*, 1994; Benbella and Paulsen, 1998), rice (Fahad *et al.*, 2016*b*), and maize (Ruiz-Vera *et al.*, 2015).

Photosynthate availability and transport capacity from source tissues to reproductive tissues may also affect reproductive development (see above). For example, in some maize hybrids, kernel number and kernel weight correspond to source capacity during grain filling, suggesting that these yield components may be limited by photosynthate supply even under non-stressed conditions (Cerrudo *et al.*, 2013). Therefore, detrimental effects of heat stress on leaf photosynthesis probably further impair grain development and yield where grain sink strength is high (Fig. 5). As discussed above, heat stress may also impair photosynthate transport between crop source and sink tissues (Suwa *et al.*, 2010; Bagley *et al.*, 2015). These studies emphasize the need for sufficient production of sugars through photosynthesis and maintenance of their transport, especially during heat stress. Although beyond the scope of this review, direct impacts of high temperature on reproductive structures also play a critical role in determining crop yields and will require engineering for greater tolerance to heat stress to ensure sufficient sink size for enhanced photosynthate production and transport (Barnabás *et al.*, 2008; Ruiz-Vera *et al.*, 2015; Ferguson *et al.*, 2021).

### Recent advances made at the plant level to improve understanding of temperature effects

Developing plant mechanisms to cope with heat stress is complicated by interacting climate factors and the geographical variability forecast for temperature (Long and Ort, 2010; Hoegh-Guldberg *et al.*, 2018), with heat stress responses greatly influenced by region and environmental conditions. Further, a combination of traits and agronomic manipulations determine heat stress tolerance. The determination of heat-tolerant crop ‘ideotypes’ is a challenge for plant breeders, and has driven a push to locate quantitative trait loci (QTLs) and genetic markers for photosynthetic heat tolerance (Azam *et al.*, 2014; Sharma *et al.*, 2017). While progress has been made, searching for QTLs is a substantial task, given the combination of changing variables throughout a plant life cycle and the challenges in genotyping and phenotyping large germplasm sets at different growth stages.

Plant phenotyping may provide a quicker means of detecting plant heat stress responses given recent technological advances (Furbank *et al.*, 2019; Furbank and Tester, 2011; Gao *et al.*, 2020). For example, plant temperature stress causes stomatal responses detectable with thermal imaging (Stoll and Jones, 2007; Prashar and Jones, 2014) and visible scorching and damage detectable with red–green–blue imaging (Elazab *et al.*, 2016). Photosynthetic responses are also detectable with chlorophyll fluorescence (Sharma *et al.*, 2012; Jedmowski and Brüggemann, 2015) and hyperspectral analysis (Dobrowski *et al.*, 2005). At the plant scale, recent advancements in field phenotyping have seen hyperspectral analysis used to predict photosynthetic capacity in field trials (Serbin *et al.*, 2012; Yendrek *et al.*, 2017; Silva-Perez *et al.*, 2018; Fu *et al.*, 2019, 2020; Furbank *et al.*, 2019; Meacham-Hensold *et al.*, 2019, 2020). Using these phenotyping tools to screen genetically targeted germplasm is required to target heat-tolerant traits for breeders.

Scaling from the leaf to the whole-plant level in translation of heat stress traits at a higher resolution remains an additional challenge. At the plant level, temperature responses are closely linked with irradiance profiles. Recent advances in functional and structural plant modelling (FSPM) (Vos *et al.*, 2010; Evers *et al.*, 2018) offer scope for deconstructing the relationship between irradiance gradients on whole-plant temperature profiles to pinpoint *T*_opt_ for leaves at different plant canopy layers. The greater challenge in creating heat-resistant crops is pairing whole-plant FSPM, which considers leaf-level physiology to suggest heat-tolerant plant ideotypes, with tools to phenotype for genetic heat-tolerant markers across a range of species and environmental conditions.

## Scaling from plants to ecosystem reinforces the complex relationship between temperature and photosynthesis

The effects of temperature on enzyme, leaf, and plant scales compound to impact crop photosynthesis and productivity at the ecosystem scale. This is due to the additive responses to the microclimate of all leaves and plants that make up a crop ecosystem (Bagley *et al.*, 2015). The microclimate impacts crop productivity through the effects of atmospheric turbulence and wind changing the temperature, humidity, and light environment experienced by leaves at different heights within the canopy (Cleugh, 1998). While the speed at which a cropping system can respond to changes in light can reduce ecosystem photosynthesis (Kromdijk *et al.*, 2016; Morales and Kaiser, 2020), increases in temperature are a crucial driver reducing photosynthesis and yields across the major cropping varieties (Lobell *et al.*, 2014; Asseng *et al.*, 2015; Liu *et al.*, 2016; C. Zhao *et al.*, 2017), and will be the focus of this section.

A key mechanism controlling the reduction in ecosystem photosynthesis at higher temperature is the link with atmospheric VPD (Bernacchi and VanLoocke, 2015). The amount of water vapour which air can hold at saturation (*e*_s_) increases with temperature, while the actual water vapour of air at any given time (*e*_a_) remains relatively constant, resulting in increased atmospheric VPD—the difference between *e*_s_ and *e*_a_ (Bernacchi and VanLoocke, 2015; Ficklin and Novick, 2017). Increasing atmospheric VPD has a feedback effect on plants, particularly on the stomata, whereby a drier atmosphere exerts a stronger pull on water from within leaves during photosynthesis (Lawson and Vialet-Chabrand, 2019). As discussed earlier, crops can close their stomata to conserve water, but this comes at the cost of photosynthesis, which reduces yield at the ecosystem scale if relied upon too often during the growing season.

Early lessons from FACE (Table 1) studies suggest that crop photosynthesis would be enhanced with higher [CO_2_], and water loss would decline with lower *g*_s_ (Leakey *et al.*, 2009). A recent update of the literature has confirmed that these conclusions hold for C_3_ and C_4_ crops (Ainsworth and Long, 2020). However, when FACE systems were coupled with increased temperature (T-FACE), canopy warming and periodic heat stress caused an acceleration in maize and soybean crop development and often decreased yield (Siebers *et al.*, 2015; Ruiz-Vera *et al.*, 2018), particularly when higher temperatures were coupled with water deficit (Gray *et al.*, 2016). Even without supplemental heating through experimentation, hotter and drier growing seasons reduced wheat yield grown under FACE relative to FACE-grown plants under ‘typical’ growing seasons (Fitzgerald *et al.*, 2016; Macabuhay *et al.*, 2018). However, mixed results have been reported for rice grown at elevated temperature, probably due to latitudinal differences in average temperature maxima impacting rice grown in the tropics more than at higher latitudes (Lesk *et al.*, 2016; Usui *et al.*, 2016).

**Fig. 6.**
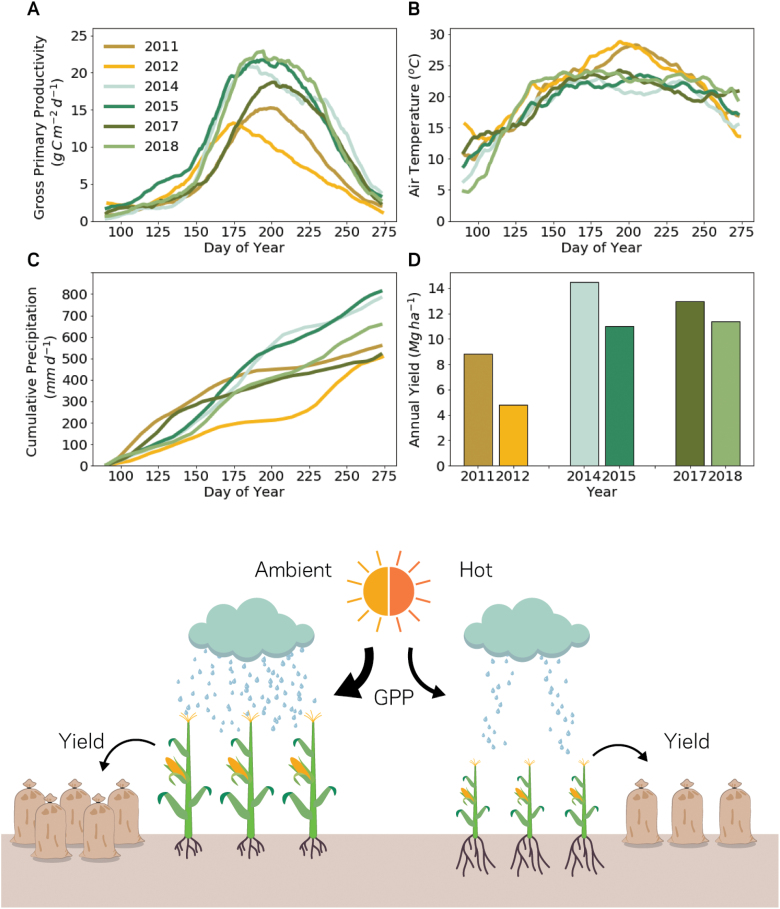
The difference in gross primary productivity (GPP) and annual yield for maize across different climatic years, as indicated by air temperature and rainfall. (A–D) were produced using data from Ameriflux site Ui-C using processing protocols from Moore *et al.* (2020). The years 2013 and 2016 are omitted from (D) as these years were under a soybean rotation at the site.

Crops grown under well-watered conditions can afford to maintain high *A* under elevated temperature for longer than crops grown under water stress (Fitzgerald *et al.*, 2016). In regions of the world where increasing temperature is coupled with increasing rainfall, drought and heat stress impacts on crop photosynthesis and productivity may be minimized (Tesfaye *et al.*, 2018). However, the timing and duration of rainfall events will be critical for determining the effectiveness of increased moisture as a buffer to hotter temperatures. For example, in the currently rain-fed and highly productive region of the Midwest United States, DeLucia *et al.* (2019) project that a water limit will be reached for maize productivity due to increased atmospheric VPD that will be driven by rising global temperature. Lobell *et al.* (2014) have shown that while maize yields have historically been increasing, the crop is very susceptible to drought and VPD stress. This impact on maize yield was evident in the 2012 drought experienced by the Midwest US during the growing season (Fig. 6). For cropping systems already reliant on irrigation, changes in mean annual rainfall associated with a warming world could be catastrophic for future yields if water resources become scarce. Shifting cropping systems that are primarily rain-fed to an irrigation-reliant system will place increased pressure on existing hydrological reserves to deliver water for agriculture in addition to metropolitan and natural systems (DeLucia *et al.*, 2019).

### Changes to the by-products of photosynthesis associated with rising temperature

Rising temperature at the ecosystem scale also affects carbon consumption processes that can impact short-term annual yield of cropping systems and their long-term ecological sustainability. For ecosystem-scale carbon cycle concepts, photosynthesis is referred to as gross primary productivity (GPP; Table 1) (Chapin *et al.*, 2006). Changes to ecosystem autotrophic respiration (RA) and GPP as global temperature increases will be likely to mirror that of the processes described earlier, in that photosynthesis has a clear *T*_opt_ and peak thermal response, and RA increases exponentially with rising temperature until acclimation occurs. However, what is less certain is the rate at which heterotrophic respiration (RH) will change as temperatures rise, particularly that of soil microbes (Bond-Lamberty and Thomson, 2010; von Haden *et al.*, 2019). It is commonly accepted that ecosystem respiration (ER; combined RA and RH) increases with temperature (Lloyd and Taylor, 1994), and can acclimate under prolonged heat exposure (Way and Yamori, 2014). A recent synthesis has suggested that this has predictably responded to global warming, though there still remains large uncertainty surrounding the RH contribution in particular (Bond-Lamberty *et al.*, 2018).

### Recent advancements and prospects for monitoring crop canopies and improving management responses with rising temperature

There is an inherent need for the development of strategies to ensure crop productivity with global warming. Current agronomic practices rely on weather and climate forecasts to predict when cropping systems are likely to require irrigation or nutrient application. However, these meteorological services lack information on real-time carbon uptake and water loss from the cropping system of interest. Such information could advance understanding of crop responses to the environment and, where possible, lead to informed management decisions to minimize losses.

Eddy covariance flux towers monitor ecosystem photosynthesis, along with water use and a suite of common meteorological measurements including air temperature, solar radiation, wind, soil moisture/temperature, and humidity (Baldocchi *et al.*, 2001). Yet, the data require large amounts of post-processing to generate complete time series for each measured variable (Isaac *et al.*, 2017; Pastorello *et al.*, 2020). Further, GPP is estimated (not measured) as the difference between the comparatively smaller net ecosystem exchange (NEE) of CO_2_ as the measured variable and ER estimated using nocturnal (Lloyd and Taylor, 1994) or diurnal (Lasslop *et al.*, 2010) temperature response functions. While this approach is imperfect in many ways, it provides the most reliable and accurate means of quantifying, with high temporal precision, the rates of photosynthesis and respiration from cropping systems at the ecosystem scale.

With >900 sites registered as part of the FLUXNET community, there still remains a paucity of flux towers providing openly available long-term monitoring data (i.e. >5 years) from agricultural systems (Baldocchi *et al.*, 2018; Cleverly *et al.*, 2020; Pastorello *et al.*, 2020). Increasing the number of flux towers operating in cropping systems in key climatic regions of the world, and making these data immediately and freely available through open-access licensing, will be an important step for improving current understanding of the wide-scale impact of rising temperature on crop ecosystem photosynthesis. The capacity to provide measurements of carbon and water fluxes in real-time is building (i.e. FluxSuite & SmartFlux from LICOR Biosciences, Lincoln, NE, USA or EasyFlux from Campbell Scientific, Logan, UT, USA), but delivering these data in real-time to land managers, as with weather forecasting, is lacking. While FLUXNET data require significant post-processing and data corrections, the end result is generally research related. Real-time output of fluxes with minimal processing may be suitable for land managers to make informed decisions. Given the link between ecosystem carbon and water fluxes, and crop photosynthetic efficiency and water stress, supplying these data in real-time would make a substantial contribution towards faster crop stress detection.

Flux tower networks also deliver important ground-truth data to validate satellite information that can be used to infer crop photosynthesis over landscape, regional, and global scales, which flux towers are incapable of completely capturing (i.e. measurement region of interest is usually between 200 m^2^ and 2000 m^2^). Satellite data products have typically relied on the calculation of vegetation indices from surface reflectance information, such as the normalized difference vegetation index (NDVI; Tucker, 1979), enhanced vegetation index (EVI; Huete *et al.*, 2002), and photochemical reflectance index (PRI; Gamon *et al.*, 1997) to provide indications of vegetation stress. However, these indices depend on changes in vegetation greenness to show variation in the index value, after which it can be too late to remedy vegetation stress. In addition, the indices typically measure top-of-canopy responses, so changes at lower canopy layers are missed.

Improvements in spectral sensing technology have led to the development of passive remote sensing of sun-induced chlorophyll fluorescence (SIF) as a proxy for real-time monitoring of photosynthesis (Meroni *et al.*, 2009; Sun *et al.*, 2017; Frankenberg and Berry, 2018). Chlorophyll fluorescence represents one of three fates of light energy absorbed by light-harvesting complexes within leaves; the other two being photochemistry and heat dissipation (Baker, 2008). Active measurement of chlorophyll fluorescence is a commonly used tool in plant physiology research, as these three light use pathways do not operate in isolation from each other. Chlorophyll fluorescence yield provides useful information on photosynthetic quantum efficiency and heat dissipation, which leads to its use in inferring *A* and in imaging to screen for genetic trait expression in plants (Murchie and Lawson, 2013). At scales from the ecosystem to globe, passive measurement of chlorophyll fluorescence as SIF relies on the spectral emission of SIF surrounding oxygen absorption bands (O_2_-A and O_2_-B) within a narrow spectral range (Meroni *et al.*, 2009; Frankenberg and Berry, 2018).

Advancements in SIF monitoring in recent years have rapidly expanded, with studies demonstrating a strong correlation between crop GPP at the ecosystem (Miao *et al.*, 2018; Wu *et al.*, 2020), regional (Guan *et al.*, 2016), and global scales (Guanter *et al.*, 2014). The relationship between SIF and crop GPP has led to the use of SIF in detecting crop stress, as the two signals are inherently linked (Zarco-Tejada *et al.*, 2012; Camino *et al.*, 2019; Peng *et al.*, 2020). Additional satellite sensing of land surface evapotranspiration (ET)—the ECOsystem Spaceborne Thermal Radiometer Experiment on Space Station (ECOSTRESS)—is also being used to assess ecosystem stress on daily time scales (Fisher *et al.*, 2020). The combination of SIF and ECOSTRESS satellite products has the potential to greatly advance our understanding of ecosystem GPP in relation to ET, and how environmental stresses, such as increased temperature and heatwaves, are likely to impact crop productivity at regional to global scales. Granted, there still remain several unanswered questions surrounding the quantity of information provided by SIF, whether the signal is primarily affected by changes in canopy architecture or if it is a direct product of biochemistry (Magney *et al.*, 2020). As these fundamental questions are answered, and with the addition of new satellite remote-sensing platforms to monitor SIF globally at high temporal resolution (i.e. TROPOMI, OCO-2, and GOME), SIF will certainly continue to advance as an important real-time tool for monitoring crop photosynthesis and productivity as global temperature rises.

## Conclusion and future directions

This review provides a comprehensive evaluation of current understanding on how crop photosynthesis responds to temperature from the enzyme to ecosystem scale. The key conclusions for each scale are summarized as follows.

(i)  Direct impacts of elevated temperature on photosynthetic enzymes involved in carbon assimilation are particularly damaging to C_3_ crops. Enzyme rates increase with temperature, but substrate specificity declines in the carbon-fixing enzyme Rubisco, which deactivates past optimal temperatures.
(ii)    Stomata typically respond to temperature through the complex effects of heat on photosynthesis, VPD, transpiration, and plant water status. Stomatal conductance can change under temperature stress, and stomatal density and size can be altered if a plant develops under hotter conditions.
(iii)  Photosynthate allocation from sources to sinks is impacted by heat stress through differential expression and activity of enzymes involved in sucrose transport and metabolism, as well as phloem structural changes.
(iv)   At the whole-plant scale, leaf interactions create temperature gradients, and heat stress impairs plant development processes.
(v)    The factors identified in (i)–(iv) act together to impact crop ecosystem photosynthesis and its response to temperature, the effects of which are typically seen as a cumulative response through the growing season and lead to reduced yield.

Ensuring our cropping systems remain resilient to rising temperatures will require integration of knowledge and information across scales. For each scale discussed, the areas of research needed to improve resiliency of cropping systems to rising temperature and heat stress are as follows.

(i)  At the biochemical scale, most strategies for improving carbon fixation in a warmer climate involve enhancing Rubisco performance or minimizing the energy expended in photorespiration, but many remain to be tested in crop species or replicated field trials.
(ii)    Altering stomatal anatomy and metabolism may help to reduce water loss from crops whilst maintaining photosynthetic rates to ensure high crop yields are maintained. However, the relationship between stomata and leaf hydraulic capacity should also be considered to maintain a balance between leaf water supply and demand.
(iii)  At the transport system level, strategies need to be tested to help maintain photosynthate allocation from sources to sinks by increasing sucrose phloem loading in sources (e.g. increasing expression of leaf sucrose transporters) and sucrose phloem unloading in sinks (e.g. increasing invertase activity in reproductive sinks), as well as increasing remobilization of sugars stored in intermediate sinks.
(iv)  Coupling whole-plant modelling of temperature gradients with phenotyping resources will allow identification and breeding of heat-resistant crop ideotypes.
(v)    At the ecosystem scale, the implementation of faster crop stress detection systems will be critical for applying management strategies to combat temperature-related stress. These strategies may include combining ground-based measurements, such as those from flux towers, with satellite remote-sensing information, to provide closer to real-time monitoring of crop systems.
